# Antioxidant activity and antibacterial evaluation of new thiazolin-4-one derivatives as potential tryptophanyl-tRNA synthetase inhibitors

**DOI:** 10.1080/14756366.2019.1596086

**Published:** 2019-04-02

**Authors:** Anca Stana, Dan C. Vodnar, Gabriel Marc, Daniela Benedec, Brînduşa Tiperciuc, Radu Tamaian, Ovidiu Oniga

**Affiliations:** a Department of Pharmaceutical Chemistry, “Iuliu Haţieganu” University of Medicine and Pharmacy, Cluj-Napoca, Romania;; b Department of Food Science and Technology, University of Agricultural Sciences and Veterinary Medicine, Cluj-Napoca, Romania;; c Department of Pharmacognosy, “Iuliu Haţieganu” University of Medicine and Pharmacy, Cluj-Napoca, Romania;; d ICSI Analytics Department, National Research and Development Institute for Cryogenics and Isotopic Technologies - ICSI Rm. Vâlcea, Râmnicu Vâlcea, Romania;; e SC Biotech Corp SRL, Râmnicu Vâlcea, Romania

**Keywords:** Thiazolin-4-one, antibacterial activity, antioxidant activity, tryptophanyl-tRNA synthetase, docking

## Abstract

The rapid emergence of bacterial resistance to antibiotics currently available for treating infectious diseases requires effective antimicrobial agents with new structural profiles and mechanisms of action. Twenty-three thiazolin-4-one derivatives were evaluated for their antibacterial activity by determining the growth inhibition zone diameter, the minimum inhibitory concentration (MIC), and the minimum bactericidal concentration (MBC), against gram-positive and gram-negative bacteria. Compounds **3a–c**, **3e–h**, **6b–c** and **9a–c** expressed better MIC values than moxifloxacin, against *Staphylococcus aureus*. Compounds **3h** and **9b** displayed similar effect to indolmycin, a tryptophanyl-tRNA ligase inhibitor. Due to their structural analogy to indolmycin, all compounds were subjected to molecular docking on tryptophanyl-tRNA synthetase. Compounds **3a–e**, **6a–e**, **8** and **9a–e** exhibited better binding affinities towards the target enzymes than indolmycin. The antioxidant potential of the compounds was evaluated by four spectrophotometric methods. Thiazolin-4-ones **3e**, **6e** and **9e** presented better antiradical activity than ascorbic acid, trolox and BHT, used as references.

## Graphical abstract

**Figure:**
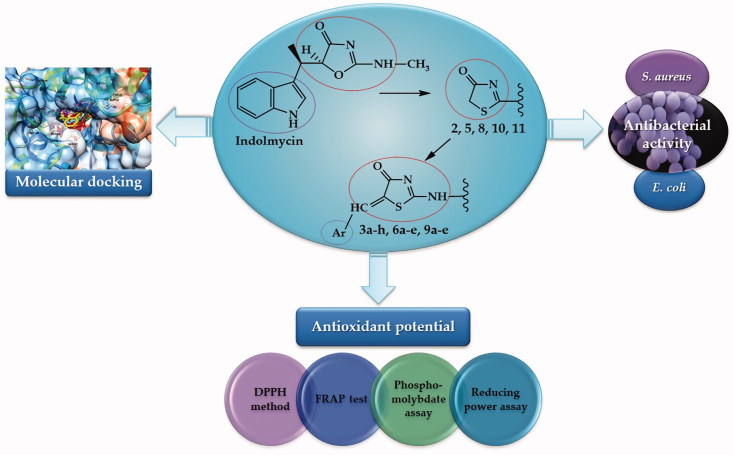

## 1. Introduction

Thiazoles, in general, and their derivatives, thiazolin-4-ones, in particular, are an important class of heterocyclic compounds, with a remarkable medicinal value. They have been associated with a large variety of significant pharmacological activities, such as antifungal^1–3^, antibacterial^4^ ^,^ ^5^, antitumor^3^ ^,^ ^4^ ^,^ ^6^, antiviral^7^, antioxidant^6^ ^,^ ^8^, neuroprotective^9^, analgesic, anti-inflammatory^4^ ^,^ ^10^ and anticonvulsant^9^.

The significant progress of medicinal chemistry, represented by the discovery of antibiotics, during the past decades led to major improvements in the diagnosis, prognosis and therapy of infections. Despite the numerous antibacterial agents approved for the treatment of infectious diseases, the continuously developing resistance of bacteria to most common classes of antibiotics and the emerging difficulties in dealing with infections outcome in hospitalised patients or in patients with impaired immune system^11^, drew the scientists’ attention towards the discovery of effective antimicrobial agents, with new structural profiles and mechanisms of action.

The aminoacyl-tRNA synthetases, also called aminoacyl-tRNA ligases, are a class of validated enzymatic targets that remain underexploited and that play an important role in RNA translation, the expression of genes to create proteins. Aminoacyl-tRNA synthetases are responsible for the precision of ribosomal protein biosynthesis by ensuring that amino acids are correctly esterified to their corresponding tRNA molecules. In general, there is a specific aminoacyl-tRNA synthetase available for each amino acid^12^. Compounds that can selectively inhibit these bacterial enzymes without interfering with their mammalian analogues are therefore potential candidates for antimicrobial agents. There have been reported several natural aminoacyl-tRNA synthetase inhibitors, like indolmycin (a tryptophanyl-tRNA synthetase (TrpRS) inhibitor), granaticin (a leucyl-tRNA synthetase inhibitor), mupirocin (a isoleucyl-tRNA synthetase inhibitor, clinically used as a topical antibacterial agent), and ochratoxin A (a phenylalanyl-tRNA synthetase inhibitor)^13^.

**Figure 1.**
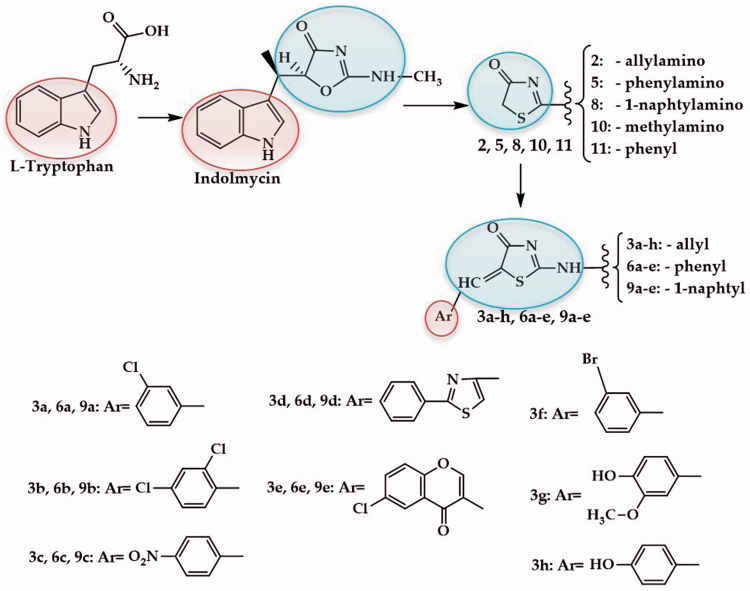
The study design and the chemical structures of the synthesized compounds.

Indolmycin is a natural antibiotic that was first isolated in 1960 and that competitively inhibits the bacterial TrpRS, due to its analogy to L-tryptophan (Figure 1). This enzyme activates L-tryptophan for translation by forming a tryptophanyladenylate intermediate and then it links this activated amino acid to the corresponding tRNA molecule (tryptophanyl-tRNA). Although potent antibacterial activity against bacteria like *Helicobacter pylori*, *Escherichia coli*, *Bacillus subtilis*, and mupirocin and methicillin-resistant *Staphylococcus aureus* was reported for indolmycin, because of its insufficient activity against common pathogenic bacteria, the development of indolmycin for chemotherapeutic use was abandoned^14^ ^,^ ^15^. Better antibacterial potential was reported against gram-positive bacteria due to a higher intracellular uptake of indolmycin by the uptake systems for tryptophan. A poorer antibacterial activity on gram-negative bacteria was attributed to the lower penetrability of indolmycin through the hydrophilic barrier of the outer membrane, due to its increased hydrophobicity^16^.

The excess of reactive oxygen and nitrogen species causes oxidative stress, that has been increasingly recognised in the last decades as an important contributing factor in the pathogenesis of many serious diseases, such as atherosclerosis, heart failure, myocardial infarction, diabetes and its complications, several neurodegenerative diseases, cancer and cirrhosis of the liver^17^. Some phenolic synthetic antioxidants, like butylhydroxytoluene and butyllhydroxyanisole, widely used as antioxidants and preservatives in the food industry, pharmaceutical preparations, and cosmetic formulations are anticipated to be tumour promoters, based on reported evidence of carcinogenicity from studies in experimental animals^18^. Therefore, there is a great demand for the discovery of new potent antioxidant therapeutics, with a better pharmaco-toxicological profile. Compounds bearing chromone, thiazole, thiazolin-4-one, or phenol moieties^8^ ^,^ ^19^ ^,^ ^20^ have been reported to possess antioxidant activities.

There is also an increased need for the discovery of novel antibacterial agents, especially for the treatment of chronic infections such as mucoviscidosis, a genetic disease that is frequently associated with infections caused by drug-resistant pathogens and epithelial damage due to pulmonary oxidative stress. In these situations, it would be useful to develop bioactive compounds that have antioxidant and antibacterial properties combined in the same molecule. A better therapeutic solution for treating complex multigenic diseases like the one mentioned above would be the development of new dual-active antibacterial-antioxidant agents^21^.

Based on the various biological activities of the thiazoline-4-ones that have been reported in the literature, we present herein the antibacterial and antioxidant activity evaluation of previously synthesized thiazoline-4-ones^2^ diversely substituted in positions 2 and 5. In order to establish the compounds potential mechanism of action, due to their structural analogy to indolmycin (Figure 1), the previously reported thiazolin-4-one derivatives^2^ were docked against two bacterial tryptophanyl-tRNA ligases and their affinities towards these biological targets were assessed. As some of the compounds have other chromophores in their structure, like the chromone, thiazole and phenol moiety, with proven antioxidant activity^8^ ^,^ ^19^ ^,^ ^20^, the antioxidant potential of the compounds was evaluated by assessing the DPPH^•^ radical scavenging activity, the ferric reducing antioxidant power (FRAP), the reducing power and the total antioxidant capacity (TAC).

## 2. Materials and methods

### 2.1. Antibacterial activity assays

Stock solutions (1 mg/mL) were prepared by dissolving the test compounds (the thiazolin-4-one derivatives and indolmycin) and the reference antibacterial agent (moxifloxacin) in sterile dimethyl sulfoxide (DMSO). Moxifloxacin and DMSO were purchased from Merck (Darmstadt, Germany) and indolmycin was purchased from Toronto Research Chemicals (North York, Canada).

The microorganisms used for the antimicrobial activity evaluation were obtained from the University of Agricultural Sciences and Veterinary Medicine Cluj-Napoca, Romania. The inhibition zone diameters, the minimum inhibitory concentration (MIC) and minimum bactericidal concentration (MBC) values were determined against cultures of gram-positive bacteria *S. aureus* ATCC 49444 and gram-negative bacteria *E. coli* ATCC 25922.

#### 2.1.1. Determination of inhibition zone diameters

The *in vitro* antimicrobial activity was determined using the cup-plate agar diffusion method according to the Clinical and Laboratory Standards Institute (CLSI) guidelines^22^.

For antibacterial testing, Mueller–Hinton agar medium and 0.5 mg/mL methylene blue (providing a better definition of the inhibition zone diameter) were used. The inoculum was prepared by suspending five representative colonies, obtained from an 18–24 h culture on nonselective nutritive agar medium, in sterile distilled water. The cell density was adjusted to the density of a 0.5 McFarland standard by measuring the absorbance in a spectrophotometer at a wavelength of 530 nm and adding sterile distilled water as required (corresponding to a population of 1–5 × 10^3^ CFU/mL). A sterile swab was soaked in suspension and then the Mueller–Hinton agar plates were inoculated by streaking the entire surface. After drying for 10–15 min, six-millimeter diameter wells were cut from the agar using a sterile cork-borer, and a volume of 20 µL of each compound solution (1 mg/mL in DMSO) was delivered into the wells (20 µg/well). Moxifloxacin (20 µg/well) was used as standard drug. The controls were performed with only sterile broth, overnight culture and 20 µL of DMSO. The plates were incubated at 35 °C. Zone diameters were measured to the nearest whole millimeter, at a point in which there was no visible growth after 24–48 h. Results were obtained in triplicate. The solvent used for the preparation of each compound stock solution (1 mg/mL), DMSO exhibited no inhibitory activity against the tested bacterial strains.

#### 2.1.2. Determination of MIC and MBC values

Antibacterial activity was tested by the broth microdilution method according to the Clinical and Laboratory Standard Institute (CLSI) guidelines^23^, following a previously reported protocol^24^. The growth control, sterility control and control of antibacterial compounds were used. The MIC was defined as the lowest concentration required for arresting the growth of bacteria. The MBC was defined as the lowest concentration of the agent at which no colonies are observed. All MIC and MBC assays were repeated three times.

### 2.2. Antioxidant activity assays

1 mg/mL stock solutions of the tested compounds (**2**, **3a–h**, **5**, **6a–e**, **8**, **9a–e**, **10** and **11**) were prepared by dissolving the solid powders in DMSO.

Overlapping of the absorption spectra of the compounds in the region 430–700 nm (Figure S1 in Supplementary Materials) was realised using a UV-VIS spectrophotometer Jasco V-530 (Jasco International Co., Tokyo, Japan) and 2.5 mL cuvettes of poly(methyl methacrylate) with a 10 mm light path. None of the tested compounds have absorption peaks near the wavelengths where the antioxidant and antiradical assays were performed (517 nm, 593 nm, 695 nm, and 700 nm). All the assays were performed in triplicate, mean values of different measurements being reported.

Analytical grade methanol and DMSO were purchased from Sigma (St. Louis, MO), DPPH^•^, ascorbic acid, trolox and dibutylhydroxytoluene (BHT) were purchased from Alfa-Aesar (Karlsruhe, Germany).

#### 2.2.1. The DPPH^•^ scavenging activity

The antioxidant potential of the synthesized compounds was assessed on the basis of their radical scavenging effect of the stable 2,2-diphenyl-1-picrylhydrazyl (DPPH^•^) following a previously described method in the literature with a few minor modifications^25^ ^,^ ^26^. The samples were dissolved in DMSO or absolute methanol according to the compound’s solubility to give a stock solution of 5 mg/mL. For the most active compounds (**3e**, **6e**, **9e**), dilutions were made to obtain a stock solution of 0.2 mg/mL. To different volumes (0.25 mL, 0.50 mL, 0.75 mL, 1 mL, 1.25 mL, 1.50 mL, 1.75 mL, 2 mL) of each compound’s stock solution, methanol was added in order to obtain 2 ml of sample solution and subsequently, 2 ml of DPPH^•^ solution in methanol (0.1 mg/mL) was added. The mixture was shaken vigorously and left to stand in dark for 30 min in a water bath at 40 °C, and then the absorbance at 517 nm was read using a UV-VIS spectrophotometer Jasco V-530 (Jasco International Co.), against a blank solution. Ascorbic acid, trolox, and BHT were used as standard antioxidants. The ability to inhibit the free radicals of DPPH^•^ was calculated according to the formula:

$$\text{Inhibition}\;\text{ratio}\%=\left[\left(A_{0}-A_{S}\right)/A_{0}\right]\times 100$$

A_0_ is the absorbance of DPPH^•^ methanol solution and A_S_ is the absorbance of sample after 30 min. The radical scavenging ability was measured as a decrease in the absorbance of DPPH^•^. Lower absorbance of the reaction mixture has indicated higher free radical scavenging activity.

Subsequently, the ability to neutralise the DPPH^•^ radicals was evaluated as a percentage, through its graphic representation according to the concentration, which allowed calculation of the EC_50_ values, the test compound’s concentration at which 50% of the DPPH^•^ radicals are scavenged. The EC_50_ value is correlated to the antioxidant capacity of the compound. The smaller this value is, the higher is the antioxidant activity of the compound. All determinations were performed in triplicate and the average thereof was the final value.

#### 2.2.2. The FRAP test

The reducing power of the tested compounds was determined by the FRAP assay, according to a modified method, initially proposed by Benzie and Strain^27–29^. 50 µL of each compound’s solution (1 mg/mL in DMSO) was mixed with 1000 µL of FRAP reagent^27^ ^,^ ^29^ and the resulted mixture were vigorously shaken for 30 min. Their absorbance was measured at *λ* = 593 nm, compared to a blank sample, where 50 µL of DMSO and 1000 µL of FRAP reagent were taken. The reducing power was compared to trolox, BHT and ascorbic acid, used as standard antioxidants and expressed as percent of control’s reducing power, based on the following formula:

% of control ferric reducing power=[(sample absorbance)/(control absorbance)]×100

A calibration curve (*R* ^2^ = 0.9993) was made using an aqueous 1 mM ferrous sulfate solution, with concentrations ranging from 0.1 to 1.0 mM, tested in the same way as the test compounds. The resulted absorbance of each tested compound was interpolated to give the equivalent Fe^2+^ amount, generated by the reduction of ferric ions.

#### 2.2.3. The phosphomolybdate assay for TAC

To determine the TAC of the tested compounds, we used a previously reported procedure^29–31^, with slight modifications. To 100 µL of each compound’s solution (1 mg/mL in DMSO), 1 mL reagent^29^ was added in test tubes, then they were well mixed and incubated for 90 min in a water bath at 95 °C. After cooling at room temperature, the absorbance of the samples was measured against blank at *λ* = 695 nm. The TAC of compounds was expressed as percent of standard antioxidant power.

% of control antioxidant power=[(sample absorbance)/(control absorbance)]×100

#### 2.2.4. The reducing power assay

In this assay, the tested compound reduces the ferric ions from potassium ferricyanide and the resulted ferrocyanide forms a blue complex in the presence of ferric ions. The principle driving this method is based on increasing the absorbance in the final test tubes. An increase in the antioxidant activity is correlated with an increase in the absorbance. The current assay was adapted to a semi-microscale based on previous literature reports^29^. 0.1 mL of the stock solutions of compounds **2**, **3a–h**, **5**, **6a–e**, **8**, **9a–e**, **10** and **11** subjected to testing, was mixed with 1 mL of DMSO, 0.4 mL of phosphate buffer (0.2 M, pH = 6.6) and 0.4 mL of K_3_[Fe(CN)_6_] (1% *w*/*v*). The mixture was incubated in a water bath at 50 °C for 20 min. After cooling at room temperature, 0.5 mL of trichloroacetic acid (10% *w*/*w*) was added. The resulting mixture was left to rest for 30 min, for eventually resulting precipitates to deposit on the bottom of the test tubes. 0.25 mL of the solution was collected carefully and mixed with 0.14 mL of FeCl_3_ (0.1% *w*/*v*) and 0.75 mL of distilled water. The absorbance was measured at *λ* = 700 nm against a blank sample. Results were calculated using the formula:

% of control reducing power=[(sample absorbance)/(control absorbance)]×100

### 2.3. Molecular docking studies

The three-dimensional structures of compounds **2**, **3a–h**, **5**, **6a–e**, **8**, **9a–e**, **10**, **11** and indolmycin, considered as ligands in the molecular docking study, were prepared based on a previously reported protocol,^32^ following the drawing and energy minimisation, carefully observing the double bond isomerism in position 5 of the thiazolin-4-one nucleus and subsequently converting to pdbqt file using OpenBabel 2.3.2^33^ and Python preparation ligand script version 4 from AutoDockTools 1.5.6^34^.

Two structures of TrpRS were used as targets in the molecular docking study: 5V0I and P67592_1I6K. The structure 5V0I, isolated from *E. coli,* was taken as it is from Protein Data Bank (http://www.rcsb.org), while P67592_1I6K was built by homology modelling, due to the lack of deposited high-resolution structures of TrpRS isolated from *S. aureus*. Based on the amino acid sequence of the *S. aureus* enzyme taken from Uniprot (P67592 sequence) and the PDB 1I6K as template, the P67592_1I6K structure was modelled using the SWISS-MODEL service^35^ ^,^ ^36^.

In the target file structures, polar hydrogen atoms were added, nonpolars were removed, ligands and other additional molecules removed, and Gasteiger charges were defined using AutoDockTools 1.5.6. Both proposed structures as targets were subjected to an analysis with BLASTP 2.6.1 service^37^, which detected conserved domains in our structures and confirmed the membership of the two structures to the PRK12282 superfamily. More, the active residues in the catalytic sites of both proteins were identified and are depicted with red in Figures S2 and S5 (in Supplementary Materials) generated after sequence alignments using EMBOSS Stretcher (https://www.ebi.ac.uk). Analysis of the pockets in the catalytic sites of the enzymes was performed using DoGSiteScorer^38^.

For both enzymes, search space was configured, taking care that all amino acids presented as important in the catalytic site were included. Search space sides were set to *x* = *y*=*z* = 60 for both targets and the centre of the search space was defined as *x* = 15.898, *y* = 6.481, *z* = 9.892 for 5V0I and *x* = 55.048, *y* = 21.595, *z* = 40.051 for P67592_1I6K. Fifty conformations were generated for each molecular docking study, clustered in 2 Å cluster tolerance. Based on the computed binding affinity (BA) energy (Δ*G*), inhibition constants (*K*_i_) were calculated using the formula:

$$K_{i}=e^{\frac{\Delta G\times 1000}{R\times T}}$$

where *R* represents the Regnault constant = $1.98719\frac{\text{kcal}}{K\times \text{mol}}$ and *T* = 298.15 K.

Visualisation and analysis of the docking results were performed using Chimera 1.10.2^39^ and AutoDockTools 1.5.6. Hydrophobicity surface representations were generated with transparency set to 50%.

## 3. Results and discussion

### 3.1. Antibacterial activity

#### 3.1.1. Determination of inhibition zone diameters

All the synthesized compounds were initially subjected to *in vitro* antimicrobial screening using the cup-plate agar diffusion method, against a gram-negative bacterial strain (*E. coli* ATCC 25922) and a gram-positive bacterial strain (*S. aureus* ATCC 49444).

The results of the antimicrobial activity testing of the 2-substituted-thiazolin-4-ones **2**, **5**, **8**, **10**, **11**, of the 2-(allyl/aryl-amino)-5-arylidene-thiazolin-4-ones **3a–h**, **6a–e**, **9a–e**, and indolmycin, in comparison with those of the reference compound used (moxifloxacin) are given in Table 1.

**Table 1.** The inhibition zone diameters of the synthesized thiazolin-4-one derivatives^a^.

Compound	*S. aureus* ATCC 49444		*E. coli* ATCC 25922	
	Diameter (mm)	%AI	Diameter (mm)	%AI
**2**	6	33.3	14	51.8
**3a**	14	77.7	14	51.8
**3b**	16	88.8	14	51.8
**3c**	16	88.8	14	51.8
**3d**	12	66.6	14	51.8
**3e**	18	100	16	59.2
**3f**	18	100	20	74.1
**3g**	18	100	16	59.2
**3h**	18	100	16	59.2
**5**	6	33.3	14	51.8
**6a**	12	66.6	16	59.2
**6b**	14	77.7	16	59.2
**6c**	16	88.8	16	59.2
**6d**	12	66.6	16	59.2
**6e**	14	77.7	16	59.2
**8**	6	33.3	18	66.6
**9a**	**20**	111.1	16	59.2
**9b**	**20**	111.1	22	81.4
**9c**	18	100	14	51.8
**9d**	14	77.7	14	51.8
**9e**	16	88.8	18	66.6
**10**	8	44.4	22	81.4
**11**	6	33.3	14	51.8
Indolmycin	**20**	111.1	14	51.8
Moxifloxacin	**18**	**100**	**27**	**100**

a All determinations were performed in triplicate (*n* = 3) and the average thereof was the final value. The values obtained for the most active compounds are marked in bold. %AI: percentage activity index [(Inhibition zone diameter of synthetic compound/Inhibition zone diameter of moxifloxacin) × 100].

All the tested compounds were active and showed moderate to good inhibitory activity against gram-positive and gram-negative bacteria. All the synthesized compounds and indolmycin were active, recording a moderate antibacterial activity against *E. coli* *ATCC* *25922* (14–22 mm inhibition zone diameters), lower than the reference antibiotic. Although no major differences were observed between the compounds’ inhibitory activities against the gram-negative bacterial strain used for testing, the thiazolin-4-ones **3f**, **8**, **9b**, **9e** and **10** were slightly more active than the rest of the compounds.

The synthesized thiazolin-4-ones and indolmycin were also active against *S. aureus* ATCC 49444, showing modest to good inhibitory activity. Of these, compounds **3e–h**, **9a–c** and indolmycin exhibited similar or better antibacterial activities than moxifloxacin. The 2-(1-naphthylamino)-5-arylidene-thiazolin-4-ones **9a–e** were, in general, more active against *S. aureus* ATCC 49444 than the thiazolin-4-ones **3a–e** and **6a–e**, suggesting that the presence of a voluminous fragment, the α-naphthyl-amine, in position 2 of the thiazolin-4-one ring is more favourable to the antibacterial activity against the gram-positive bacterial strain, than the presence of an allylamine or phenylamine fragment, probably as a result of some differences regarding the compounds’ intracellular uptake. The 2-(allyl/aryl-amino)-thiazolin-4-ones **2**, **5** and **8** presented lower inhibitory activities than the 5-substituted derivatives, indicating that the introduction of an arylidene rest in position 5 of the thiazolin-4-one moiety significantly increases the antibacterial potential against *S. aureus* ATCC 49444.

Also, the fact that compound **3f** (having a 3-bromobenzylidene moiety in its structure) was more active than compound **3a** (with a 3-chlorobenzylidene rest) against both bacterial strains tested, suggested that the presence of a more voluminous halogen atom, like bromine, in position 3 of the benzene ring enhances the antibacterial properties of the compound. Supplementary, compounds bearing two chlorine atoms were generally more active than the compounds with only one chlorine atom (compounds **b** vs. compounds **a**), suggesting that the presence of more halogen atoms in the molecule is favourable to the antibacterial activity (probably due to an increase in compound’s lipophilicity that enhances the intracellular uptake of the compound by the bacteria).

#### 3.1.2. Determination of MIC and MBC values

Prompted by the results obtained in the antimicrobial screening using the agar diffusion method, MICs and MBCs were determined, employing the broth microdilution method. All the synthesized compounds and indolmycin were tested against the same bacteria strains used in the previous study (*S. aureus* ATCC 49444 and *E. coli* ATCC 25922). Moxifloxacin was used as positive control for antibacterial activity. The results are depicted in Table 2.

**Table 2.** Minimum inhibitory concentration – MIC (in μg/mL) and minimum bactericidal concentration – MBC (in μg/mL) of thiazolin-4-one derivatives^a^.

Samples	*S. aureus* ATCC 49444			*E. coli* ATCC 25922		
	MIC	MBC	MBC/MIC ratio	MIC	MBC	MBC/MIC ratio
**2**	62.5	**62.5**	1	62.5	125	2
**5**	62.5	125	2	62.5	125	2
**8**	62.5	125	2	15.62	31.25	2
**10**	62.5	125	2	7.81	15.62	2
**11**	62.5	125	2	62.5	125	2
**3a**	**31.25**	**62.5**	2	62.5	62.5	1
**3b**	**31.25**	**62.5**	2	62.5	62.5	1
**3c**	**31.25**	**31.25**	1	62.5	62.5	1
**3d**	62.5	125	2	62.5	62.5	1
**3e**	**15.62**	**31.25**	2	62.5	62.5	1
**3f**	**7.81**	**15.62**	2	31.25	62.5	2
**3g**	**31.25**	**31.25**	1	62.5	125	2
**3h**	**0.97**	**1.95**	2	62.5	125	2
**6a**	62.5	125	2	62.5	125	2
**6b**	**31.25**	**62.5**	2	62.5	125	2
**6c**	**31.25**	**62.5**	2	62.5	125	2
**6d**	62.5	**62.5**	1	62.5	125	2
**6e**	62.5	125	2	62.5	125	2
**9a**	**3.9**	**7.81**	2	62.5	125	2
**9b**	**0.97**	**1.95**	2	15.62	15.62	1
**9c**	**31.25**	**62.5**	2	62.5	125	2
**9d**	125	250	2	125	250	2
**9e**	62.5	**62.5**	1	62.5	125	2
Indolmycin	**0.97**	**1.95**	2	31.25	62.5	2
Moxifloxacin	**31.25**	**62.5**	**2**	**1.95**	**3.9**	**2**
Inoculum control	+++	+++	–	+++	+++	–
Broth control	No growth	No growth	–	No growth	No growth	–

The values obtained for the most active compounds are marked in bold.

– indicates no inhibitory activity; +++ indicates growth in all concentrations.

a All determinations were performed in triplicate (*n* = 3) and the average thereof was the final value.

The MIC values against *S. aureus* ranged from 0.97 µg/mL (compounds **3h**, **9b** and indolmycin) to 125 µg/mL (compound **9d**) and the MBC values ranged from 1.95 µg/mL (compounds **3h** and **9b**) to 250 µg/mL (compound **9d**). Against *E. coli*, the compounds’ MIC values ranged from 7.81 µg/mL (compound **10**) to 125 µg/mL (compound **9d**) and the MBC values ranged from 15.62 µg/mL (compounds **10** and **9b**) to 250 µg/mL (compound **9d**). Analyzing the results obtained, it can be seen that the growth inhibitory activity was more pronounced against *S. aureus*, where 12 compounds exhibited similar or higher MIC values than moxifloxacin and 15 compounds presented similar or higher MBC values than moxifloxacin. The strain of *E. coli* was less sensitive to the activity of thiazolin-4-one derivatives, which displayed lower MIC and MBC values than the antibacterial used as a reference, in agreement with the inhibitory zone diameters.

All the synthesized thiazolin-4-ones presented moderate to good antibacterial properties. Overall, the compounds were more active against the gram-positive bacterial strain than against the gram-negative bacterial strain used in the antibacterial activity assays. The thiazolin-4-one derivatives **3h** and **9b** displayed the best antibacterial activity against *S. aureus*, similar to indolmycin and 32 fold better than that of moxifloxacin. The most active compounds against *E. coli* were the thiazolin-4-ones **8**, **10** and **9b**, which presented similar or better antibacterial activity than indolmycin, but lower than moxifloxacin. The calculated MBC/MIC ratio suggested a bactericidal effect for these compounds.

### 3.2. Antioxidant activity

One of the bacterial mechanisms of resistance to antibiotics is the ability to form biofilms. Oxidative stress was recently reported to be a potential contributing factor for the selection of resistant bacterial strains, since reactive oxygen species were incriminated in the selection of pro-biofilm variants selection^40^, thus the need for discovery of agents with both antibacterial and antioxidant activities.

#### 3.2.1. The DPPH^•^ scavenging activity

The antioxidant potential of all the synthesized compounds was assessed by measuring their ability to act as free radical scavengers or hydrogen donors, using the DPPH (2,2-diphenyl-1-picrylhydrazyl) method. The results of the antioxidant activity of thiazolin-4-one derivatives in comparison with those of the antioxidants (BHT, trolox, and ascorbic acid) used as references are presented in Table 3.

**Table 3.** The DPPH^•^ scavenging activity of thiazolin-4-one derivatives.

Compound	EC_50_ (μg/mL)^a^
**2**	2177 ± 1.3
**3a**	–
**3b**	–
**3c**	2747 ± 2.2
**3d**	–
**3e**	**20 ± 0.9**
**3f**	–
**3g**	2524 ± 1.1
**3h**	2498 ± 1.5
**5**	590 ± 1.0
**6a**	2604 ± 1.2
**6b**	–
**6c**	2634 ± 1.6
**6d**	–
**6e**	**12 ± 0.8**
**8**	–
**9a**	4685 ± 1.9
**9b**	–
**9c**	885 ± 2.0
**9d**	> 5000
**9e**	**24 ± 1.4**
**10**	665 ± 0.7
**11**	923 ± 1.3
BHT	**47 ± 0.5**
Trolox	**65 ± 1.2**
Ascorbic acid	**25 ± 0.3**

a Mean ± SD (*n* = 3).– indicates no free radical scavenging activity. The values obtained for the most active compounds are marked in bold.

Of the 23 thiazolin-4-one derivatives tested, 15 showed a modest to good inhibitory activity, the rest being inactive compounds. The most active compounds were the thiazolin-4-one derivatives **3e**, **6e** and **9e**. They exhibited better antioxidant activity than the reference compounds used (ascorbic acid, trolox and BHT), suggesting that the presence of a chromonyl rest in their structure enhances the antiradical activity of the compounds by increasing their capacity to act as hydrogen donors.

#### 3.2.2. The reducing power by FRAP test

The electron donating capacity of thiazolin-4-one derivatives was determined spectrophotometrically using the FRAP assay. This assay is based on the reduction of ferric ions to ferrous ions by the tested molecules. The resulted ferrous ions form a blue-coloured complex (Fe^2+^-TPTZ) at pH = 3.6 with tripyridyltriazine (2,4,6-Tris(2-pyridyl)-*s*-triazine). The amount of blue complex resulted is proportional with the reducing capacity of Fe^3+^ by the tested compounds. The results obtained are presented in Table 4.

**Table 4.** The ferric reducing capacity of thiazolin-4-ones by FRAP test^a^.

Compound	% of trolox reducing power (mg/mg)	% of ascorbic acid reducing power (mg/mg)	% of BHT reducing power (mg/mg)	Fe^2+^ equivalents generated
**2**	5.97	6.21	8.87	0.1221
**3a**	5.73	5.96	8.51	0.1183
**3b**	7.93	8.25	11.78	0.1536
**3c**	9.51	9.90	14.13	0.1790
**3d**	5.77	6.01	8.57	0.1189
**3e**	**24.61**	**25.61**	**36.55**	**0.4214**
**3f**	12.76	13.28	18.96	0.2312
**3g**	11.98	12.47	17.79	0.2186
**3h**	7.94	8.26	11.79	0.1538
**5**	7.74	8.05	11.49	0.1505
**6a**	16.20	16.86	24.06	0.2864
**6b**	5.52	5.75	8.20	0.1149
**6c**	7.23	7.53	10.74	0.1424
**6d**	7.75	8.07	11.51	0.1507
**6e**	**35.86**	**37.32**	**53.26**	**0.6022**
**8**	5.84	6.08	8.68	0.1200
**9a**	8.16	8.50	12.13	0.1573
**9b**	5.07	5.28	7.53	0.1076
**9c**	9.59	9.99	14.25	0.1803
**9d**	10.12	10.54	15.04	0.1888
**9e**	**46.15**	**48.03**	**68.55**	**0.7675**
**10**	10.04	10.45	14.00	0.1875
**11**	16.79	17.47	24.93	0.2958
Trolox	N/A	N/A	N/A	1.6323
Ascorbic acid	N/A	N/A	N/A	1.5694
BHT	N/A	N/A	N/A	1.1076

N/A: not available/assigned. The values obtained for the most active compounds are marked in bold.

a All determinations were performed in triplicate (*n* = 3) and the average thereof was the final value.

All the compounds presented lower reducing capacities than those of the reference antioxidants (trolox, ascorbic acid, and BHT) used in the assay. The compounds with the best reducing abilities were the thiazolin-4-one derivatives **3e**, **6e** and **9e** that have a chromonyl moiety in their structure.

#### 3.2.3. The phosphomolybdate assay for TAC

The assay is based on the reduction of Mo^6+^ to Mo^5+^ in the presence of a reducing agent (antioxidant), involving one electron transfer mechanism with the formation of a green phosphate Mo^5+^ complex at acidic pH, which can monitored spectrophotometrically at 695 nm. A higher absorbance value indicates a better antioxidant power of the compound, when compared to a standard antioxidant.

The results of the compounds’ TAC are presented in Table 5. Compounds **6a**, **6c**, **9c** and **9e** exhibited good total antioxidant capacities. The most active thiazolin-4-one derivative was **6e**, which presented a total antioxidant activity equivalent to 171.63% of BHT’s antioxidant power.

**Table 5.** The total antioxidant capacity of the thiazolin-4-one derivatives^a^.

Compound	% of trolox antioxidant power (mg/mg)	% of ascorbic acid antioxidant power (mg/mg)	% of BHT antioxidant power (mg/mg)
**2**	5.75	3.34	17.69
**3a**	5.13	2.97	15.76
**3b**	8.47	4.91	26.03
**3c**	10.36	6.01	31.85
**3d**	4.61	2.67	14.17
**3e**	9.48	5.50	29.13
**3f**	10.83	6.28	33.30
**3g**	10.62	6.16	32.65
**3h**	5.13	2.97	15.76
**5**	6.44	3.73	19.78
**6a**	22.51	13.05	**69.17**
**6b**	10.27	5.95	31.56
**6c**	22.69	13.16	**69.74**
**6d**	6.91	4.01	21.25
**6e**	**55.84**	**32.38**	**171.63**
**8**	5.61	3.25	17.25
**9a**	6.72	3.90	20.66
**9b**	9.01	5.23	27.70
**9c**	13.84	8.03	**42.54**
**9d**	5.93	3.44	18.21
**9e**	13.28	7.70	**40.82**
**10**	9.25	5.36	28.42
**11**	9.14	5.30	28.10

a All determinations were performed in triplicate (*n* = 3) and the average thereof was the final value. The values obtained for the most active compounds are marked in bold.

#### 3.2.4. The reducing power assay

In this assay, the reduction of ferricyanide to ferrocyanide gave the Perl’s Prussian blue, in the presence of ferric ions. The resulted blue compound has an absorption peak at *λ* = 700 nm. The absorbance measured is directly proportional with the percent of compound’s reducing power when compared to a reference antioxidant. The results obtained in this experiment are presented in Table 6. All compounds exhibited lower reducing power than the antioxidants used as standards, and of these, compounds **3e**, **6a**, **6e** and **9e** presented the best reducing properties.

**Table 6.** The reducing power of thiazolin-4-ones^a^.

Compound	% of trolox reducing power (mg/mg)	% of ascorbic acid reducing power (mg/mg)	% of BHT reducing power (mg/mg)
**2**	0.44	0.44	0.50
**3a**	2.16	2.15	2.43
**3b**	3.71	3.68	4.18
**3c**	1.13	1.12	1.27
**3d**	6.35	6.30	7.14
**3e**	**10.55**	**10.47**	**11.87**
**3f**	7.81	7.75	8.79
**3g**	1.90	1.88	2.13
**3h**	0.18	0.18	0.20
**5**	6.56	6.51	7.38
**6a**	**21.04**	**20.87**	**23.67**
**6b**	3.25	3.22	3.65
**6c**	7.59	7.53	8.54
**6d**	5.50	5.45	6.18
**6e**	**10.13**	**10.05**	**11.40**
**8**	0.87	0.86	0.98
**9a**	1.21	1.20	1.36
**9b**	3.40	3.37	3.82
**9c**	0.40	0.40	0.45
**9d**	6.72	6.66	7.56
**9e**	**17.13**	**16.99**	**19.26**
**10**	0.57	0.57	0.65
**11**	4.83	4.79	5.43

^a^All determinations were performed in triplicate (*n* = 3) and the average thereof was the final value. The values obtained for the most active compounds are marked in bold.

### 3.3. Molecular docking studies

All the investigated molecules (the thiazolin-4-one derivatives and indolmycin) were virtually subjected to docking, against the designated bacterial target (TrpRS) in order to investigate the potential binding mode and BA of these compounds. Docked ligand conformations (poses) were analyzed in terms of BA (expressed in kcal/mol) and polar interactions between the best poses and their target protein. Detailed analyses of the ligand-receptor interactions were carried out and final possible orientations of the ligands and receptors were saved.

#### 3.3.1. Docking against bacterial tryptophanyl-tRNA synthetase

Sequence alignment performed between *S. aureus* TrpRS’s primary sequence P67592 and the sequence of the template PDB 1I6K structure, used in the homology modelling, suggested a common origin of both proteins. Multiple blocks or individual conserved residues were found between these two sequences (Figure S2, in Supplementary Materials). Based on these preliminary results, we considered that with the use of these two structures, we can develop the chimeric tridimensional structure of the corresponding missing TrpRS for *S. aureus*.

There are many validated primary structures of TrpRS isolated from various strains of *S. aureus* deposited in UniProt: P67593 – (strain N315), P67592 – strain Mu50/ATCC 700699, Q5HH88 – strain COL, Q6GI89 – strain MRSA252, Q6GAT0 – strain MSSA476, P67594 – strain MW2, T1Y8L7 from *S. aureus* subsp. *aureus* CN1, A0A0E0VMI8 from *S. aureus* subsp. *aureus* 71193, A0A0E1AGS6 from *S. aureus* subsp. *aureus* Z172, Q2FZQ7 – strain NCTC 8325, A0A0E1VIN1 from *S. aureus* subsp. *aureus* USA300_TCH959, A0A0E1X8M2 from *S. aureus* subsp. *aureus* MN8, A0A0H3KFE9 – strain Newman, A0A0H2XGX6 – strain USA300, A0A2H5AMI2 - strain 046. Studying these primary structures of the enzyme it was observed that, with the exception of the last one, which has a mutation, they are identical, thus the tryptophanyl-tRNA ligase of *S. aureus* is a highly conserved protein, without mutations, that can be considered a very attractive target for new antibiotics because along the way, during evolution, has not undergone evolution’s pressure and did not develop mutations over time.

**Figure 2.**
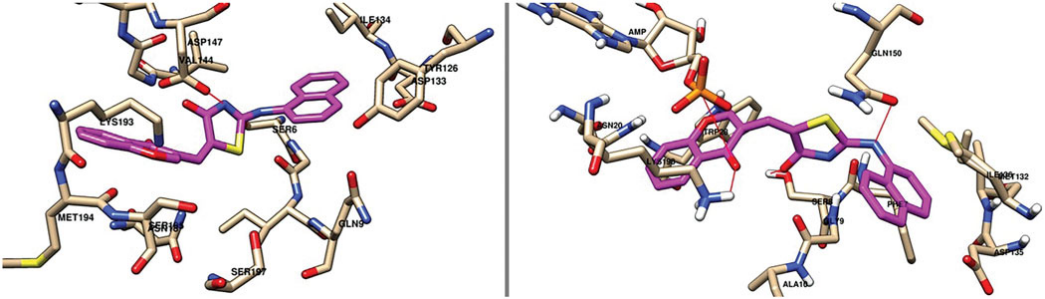
Docking pose of compound 9e in the active site of *S. aureus* TrpRS (left image) and *E. coli* TrpRS (right image) – the target is depicted as nude thin sticks, meanwhile the ligand is figured as pink sticks.

Compounds **2**, **3a–h**, **5**, **6a–e**, **8**, **9a–e**, **10**, **11** and indolmycin were docked *in silico* into the catalytic site of both TrpRSs (1I6K_P67592 and 5V0I). The predicted BA of the top binding conformation of the tested compounds to the catalytic sites of the enzymes and the consequent computed inhibition constant (*K*_i_) of the best poses are presented in Table 7. The binding manner of all compounds in the catalytic sites of both enzymes is depicted in Figure S3 (*S. aureus*) and Figure S4 (*E. coli*) in Supplementary Materials. The docking pose of the compound with the best BA toward the two targets, the thiazolin-4-one derivative **9e**, is presented in Figure 2.

**Table 7.** The top binding affinity of compounds to *E. coli* TrpRS and *S. aureus* TrpRS, the computed inhibition constant and the mean binding affinities of compounds’ conformations in the 2 Å cluster.

Compound	1I6K_P67592 (*S. aureus*)				PDB 5V0I (*E. coli*)			
	BA (kcal/mol)	*K*_i_ (nM)	2 Å cluster		BA (kcal/mol)	*K*_i_ (nM)	2 Å cluster	
			No. conf	Mean BA (kcal/mol)			No. conf	Mean BA (kcal/mol)
**2**	−5.13	173642.86	16	−4.88	−5.09	185770.75	25	−8.20
**3a**	**−7.96**	**1462.98**	21	−7.72	−8.15	1061.61	22	−7.88
**3b**	**−8.11**	**1135.76**	21	−7.92	−8.12	1116.75	14	−7.87
**3c**	**−9.33**	**144.88**	42	−8.96	−7.56	2873.70	7	−7.25
**3d**	**−9.15**	**196.32**	33	−9.10	**−8.9**	**299.37**	12	−8.42
**3e**	**−8.94**	**279.83**	31	−8.61	**−8.89**	**304.47**	2	−8.87
**3f**	**−7.77**	**2016.09**	39	−7.66	−8.42	673.06	35	−8.32
**3g**	−7.63	2553.47	5	−7.56	−8.19	992.31	36	−8.04
**3h**	−7.62	2596.94	10	−7.56	−7.86	1731.96	43	−7.81
**5**	−6.1	33778.23	47	−5.96	−6.79	10540.46	50	−6.77
**6a**	**−8.59**	**505.17**	23	−8.50	**−9.79**	**66.65**	18	−9.41
**6b**	**−8.97**	**266.01**	21	−8.68	**−9.82**	**63.36**	13	−9.37
**6c**	**−10.11**	**38.84**	28	−9.99	**−8.86**	**320.28**	10	−8.75
**6d**	**−10.57**	**17.87**	48	−10.41	**−9.33**	**144.88**	14	−9.11
**6e**	**−10.34**	**26.34**	35	−10.20	**−10.31**	**27.71**	3	−10.17
**8**	**−7.88**	**1674.47**	50	−7.83	−8.58	513.77	46	−8.46
**9a**	**−10.51**	**19.77**	33	−10.22	**−11.12**	**7.06**	32	−10.77
**9b**	**−10.82**	**11.72**	21	−10.48	**−11.84**	**2.09**	22	−11.32
**9c**	**−11.24**	**5.77**	22	−10.87	**−10.87**	**10.77**	2	−10.48
**9d**	**−11.36**	**4.71**	40	−10.97	**−10.82**	**11.72**	1	−10.82
**9e**	**−12.1**	**1.35**	37	−11.55	**−12.2**	**1.14**	3	−11.95
**10**	−5.73	63074.05	17	−5.71	−6.48	17786.60	47	−6.45
**11**	−4.34	658771.95	48	−4.33	−4.37	626245.74	35	−4.36
Indolmycin	**−7.67**	**2386.77**	5	−7.48	**−8.65**	**456.52**	25	−8.20

Bold values: The best binding affinities (higher than those of the reference compound). BA: binding affinity (expressed in kcal/mol); No. conf: number of conformations; *K*_i_: inhibition constant.

For the top binding conformation of each compound, the number of other conformations out of the 50 predicted found in the 2 Å cluster inclusion limit are presented in Table 7.

There is an obvious increase in the interaction with the biological targets for the compounds functionalised in position 5 of the thiazolin-4-one nucleus, compared to the unsubstituted ones (the thiazolin-4-ones **2**, **5**, **8** vs. the 5-arylidene-thiazolin-4-ones **3a–h**, **6a–e**, **9a–e**). The best binding energies were obtained for the compounds that have a bulky residue such as phenylamino (compounds **6a–e**) or alpha-naphthylamino (compounds **9a–e**) in position 2 of the thiazolin-4-one nucleus. The presence of a smaller residue such as allylamino leads to diminished binding energies.

An increase in the BA towards the target is found also if the residue introduced in position 5 of the thiazolin-4-one ring by Knoevenagel condensation is a bulky, binuclear one (2-phenyl-thiazolyl, chromonyl), to the detriment of mononuclear ones (phenyl or substituted phenyl).

Considering the top binding conformations of thiazolin-4-one derivatives to the active site of the TrpRSs, we further focused on specific AA binding interactions. For each compound, the main polar interactions made by the compounds’ best docking pose to the amino acids residues from the catalytic site of each enzyme are presented in Table 8.

**Table 8.** Polar contacts made by the top binding conformation of each compound to the catalytic site of TrpRS.

Compound	1I6K_P67592 (*S. aureus*)		PDB 5V0I (*E. coli*)	
	AA residue (s)	Interacting atom(s)	AA residue (s)	Interacting atom(s)
**2**	Tyr126	C=O (tz4one)	Gly9	C=O (tz4one)
	Gly7	N–H		
**3a**	Gly145	N–H	Gly9	C=O (tz4one)
			Gly147	N–H
**3b**	Asn18	N–H	Gly9	N–H
**3c**	Tyr126	S (tz4one)	Gly9 Asn20	N (tz4one) N = O
	Ile8	C=O (tz4one)		
	Lys196	C=O (carboxyl)		
	Ser195	C=O (carboxyl)		
	Lys193	C=O (carboxyl)		
**3d**	Asn18	N (th)	Gly9 PO_4 _PO_4_	N (th) S (th) N–H
	Gly145	S (th)		
	Gln9	C=O (tz4one)		
	Tyr126	N–H		
**3e**	Gly145	C–O–C (chr)	Asp135	C–O–C (chr)
			Tyr128	N–H
	Gly7	N–H		
			Gln150	S (th)
**3f**	Gly145	N–H	Gln11	N–H
**3g**	Asn18	Ph–OH	Met132 Gln11	Ph–OH N (tz4one)
	Gly145	C–O–C		
	Gly7	S (tz4one)		
	Thr126	C=O (tz4one)		
**3h**	Asn18	Ph–OH	Met132 Gln11	Ph–OH N (tz4one)
	Tyr126	N–H		
	Gly7	C=O (tz4one)		
**5**	Gln9	C=O (tz4one)	Gly9	N–H
	Thr126	S (tz4one)	Gly147	C=O (tz4one)
**6a**	Gly145	N–H	Gly9	N–H
			Gly147	C=O (tz4one)
**6b**	Gly145	N–H	Gly9	N–H
			Gly147	C=O (tz4one)
**6c**	Lys193	N = O		
	Lys196	N = O	Gly9	N–H
	Gly7	N–H	Gly147	C=O (tz4one)
	Tyr126	C=O (tz4one)		
**6d**	Gly7	N (th)	Phe7	S (th)
			Gly9	N (th)
			His45	NH
**6e**	N/A	N/A	N/A	N/A
**8**	Gln9	C=O (tz4one)	Gly147	C=O (tz4one)
			Gly9	N–H
**9a**	Gly145	C=O (tz4one)	N/A	N/A
**9b**	Asn18	N–H	Gly9	N (tz4one)
**9c**	Lys193	N = O	Gly9	N (tz4one)
	Gly7	N–H		
**9d**	Asn18	N–H	N/A	N/A
	Gly7	N (th)		
**9e**	Asn18	N–H	Gly9	N (tz4one)
**10**	Asn18	N (tz4one)	Gly9	N (tz4one)
**11**	Tyr126	C=O (tz4one)	Gly9	N (tz4one)
	Gly7	N–H		
Indolmycin	Asp133	N–H (indole)	Met132	N–H (indole)
			His45	N–H (exoc)
			Gln11	N (oz4one)

Tz4one: thiazolin-4-one; oz4one: oxazolin-4-one; th: thiazole; chr: chromone; exoc: exocyclic.

A global difference regarding the binding mode of all the compounds between the two targets has been found. Noting the significant binding differences in the active sites of the compounds tested between the two targets, both in terms of interaction and binding homogeneity, we proceeded to analyze the sequence and the catalytic sites of the two enzymes. The results of the catalytic site analysis are presented in the Figures S5 and S6, and Table T1 in Supplementary Materials. TrpRSs are known to have very flexible structures, with significantly different conformations depending on the ligand bound^13^. The homology model 1I6K_P67592 was based on a crystal structure with tryptophanyl-adenylate bound and the PDB 5V0I is a crystal structure with tryptophanyl + AMP. Besides the discrepancies between the targets’ primary sequences, the different ligands bound could also be responsible for the differences observed between the active sites of the two enzymes, the two targets having different conformational states.

## 4. Conclusions

Twenty-three previously synthesized thiazolin-4-one derivatives and indolmycin have been investigated *in vitro* for their antibacterial potential against a gram-positive (*S. aureus*) and a gram-negative (*E. coli*) bacterial strain with the help of two different assays: the cup-plate agar diffusion method and the broth microdilution method. Compounds **3a–c**, **3e–h**, **6b–c** and **9a–c** were the most active against *S. aureus*, with MIC values similar or better than those of moxifloxacin. All compounds displayed antibacterial activity against *E. coli*, but inferior to that of moxifloxacin, used as reference antibiotic. The results of the inhibition zone diameter determination and MBC values determination were in agreement with the results obtained. The MBC/MIC ratio calculated for these compounds suggested a bactericidal effect.

The possible interactions with the TrpRSs were evaluated *in silico*. The molecular docking study, performed on TrpRS from *S. aureus* and *E. coli*, revealed that the thiazolin-4-ones have a common binding pattern to the enzyme, involving polar contacts between exocyclic secondary amine group, the C = O group from the thiazolin-4-one ring with AA residues from the active site of the enzyme. Sixteen thiazolin-4-ones presented better binding affinities to 1I6K_P67592 from *S. aureus* and twelve presented better binding energies to PDB 5V0I from *E. coli* than indolmycin. The substitution of position 2 of the thiazolin-4-one ring with a voluminous moiety seemed to enhance the BAs to the target enzymes, leading to compounds that mimic better indolmycin. Further *in vitro* studies are needed to confirm that the compounds actually bind to the TrpRSs and to establish the mechanism of action of the synthesized thiazolin-4-ones.

The antioxidant potential of the thiazolin-4-ones was also evaluated by four different assays, in one of these, the radical scavenging properties were tested (the DPPH^•^ radical scavenging activity method) and the other three were based on the compounds’ ability to act as reducing agents or to participate in electron transfer reactions (the FRAP method, the reducing power assay and the phosphomolybdate assay for TAC). Compounds **3e**, **6e** and **9e** bearing a chromonyl moiety presented the best antioxidant properties, comparable to the reference antioxidants.

From all compounds synthesized, compound **9e** was the most active compound regarding both biological activities, antibacterial and antioxidant, and also displayed the best BAs towards the bacterial TrpRSs *in silico*. This suggests that the presence of an α-naphthylamino rest in position 2 of the thiazolin-4-one ring and a chromonyl moiety in the structure is favourable for obtaining dual-targeting, antibacterial and antioxidant compounds. These moieties also seem to be important for bacterial targeting, as they enhanced the BA of the molecule towards the bacterial TrpRS.

These preliminary results obtained from the *in vitro* antibacterial and antioxidant activity evaluation and the molecular docking study may serve as a starting point for designing new heterocyclic compounds with antioxidant potential and antibacterial activity, acting as TrpRS inhibitors.

## Supplementary Material

Supplemental Material
