# Supplementary material for: Antioxidant activity and antibacterial evaluation of new thiazolin-4-one derivatives as potential tryptophanyl-tRNA synthetase inhibitors
Source: J Enzyme Inhib Med Chem. 2019 Apr 2;34(1):898–908. doi: 10.1080/14756366.2019.1596086 (PMC6450493; doi:10.1080/14756366.2019.1596086)
Supplement: Supplemental Material [file IENZ_A_1596086_SM3625.pdf]

Supplementary Materials for

Antioxidant Activity and Antibacterial Evaluation of New Thiazolin-4-one Derivatives as Potential Tryptophanyl-tRNA Synthetase Inhibitors

Anca Stana[a†], Dan C. Vodnar[b]*, Gabriel Marc[a†], Daniela Benedec[c], Brînduşa Tiperciuc[a†], Radu Tamaian[d,e] and Ovidiu Oniga[a]

[a]*Department of Pharmaceutical Chemistry, "Iuliu Haţieganu" University of Medicine and Pharmacy, 41 Victor Babeş Street, RO-400012 Cluj-Napoca, Romania;* [b]*Department of Food Science and Technology, University of Agricultural Sciences and Veterinary Medicine, 3-5 Manăştur Street, RO-400372 Cluj-Napoca, Romania;* [c]*Department of Pharmacognosy, "Iuliu Haţieganu" University of Medicine and Pharmacy, 12 I. Creangă Street, Cluj-Napoca 400010, Romania;* [d]*ICSI Analytics Department, National Research and Development Institute for Cryogenics and Isotopic Technologies - ICSI Rm. Vâlcea, 4th Uzinei Street, RO-240050 Râmnicu Vâlcea, Romania;* [e]*SC Biotech Corp SRL, 4th Uzinei Street, RO-240050 Râmnicu Vâlcea, Romania*

***** Correspondence: dan.vodnar@usamvcluj.ro; Tel.: +40-264-596-386

† These authors contributed equally to this work.

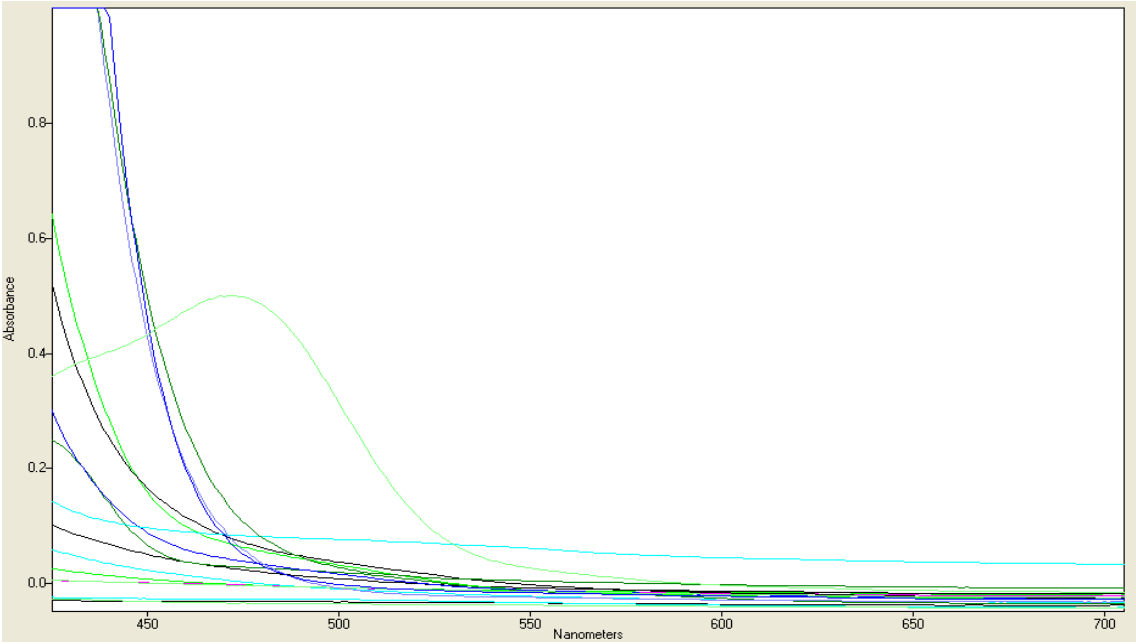

Figure S1. Overlapping of the absorption spectra of the compounds in the region 430-700 nm. None of the tested compounds have absorption peaks near the wavelengths at which the antioxidant and antiradical assays were performed (517 nm, 593 nm, 695 nm and 700 nm).

A sequence alignment was performed between *S. aureus* tryptophanyl-tRNA synthetase's primary sequence P67592 and the sequence of the template PDB 1I6K structure, used in the homology modeling (Figure S2). EMBOSS Stretcher returned the following scores as a result of the alignment between the two sequences: identity=62.3% (205/329), similarity=81.8% (269/329) and gaps=0.3% (1/329).

```
P67592   1 METLFSGIQPSGIPTIGNYIGALKQFVDVQNDYDCYFCIVDQHAITMPQD     50
           |:|:||||||||:.|||||||||:|||::|::|:||||||||||||:.||
1I6K     1 MKTIFSGIQPSGVITIGNYIGALRQFVELQHEYNCYFCIVDQHAITVWQD     50

P67592  51 RLKLRKQTRQLAAIYLASGIDPDKATLFIQSEVPAHVQAGWMLTTIASVG    100
           ..:||:..|:|||:|||.||||.:||||||||||||.||.|||..|..:|
1I6K    51 PHELRQNIRRLAALYLAVGIDPTQATLFIQSEVPAHAQAAWMLQCIVYIG    100

P67592 101 ELERMTQYKDKAQKAVEGIPAGLLTYPPLMAADIVLYNTNIVPVGDDQKQ    150
           |||||||:|:|:. ..|.:.||||||||||||||:||||:|||||:||||
1I6K   101 ELERMTQFKEKSA-GKEAVSAGLLTYPPLMAADILLYNTDIVPVGEDQKQ    149

P67592 151 HIELTRNLVDRFNSRYNDVLVKPEIRMPKVGGRVMSLQDPTRKMSKSDDN    200
           ||||||:|.:|||.||.::...||.|:||||.|:|||.|||:||||||.|
1I6K   150 HIELTRDLAERFNKRYGELFTIPEARIPKVGARIMSLVDPTKKMSKSDPN    199

P67592 201 AKNFISLLDEPNVAAKKIKSAVTDSDGIIKFDRDNKPGITNLISIYAGLT    250
           .|.:|:|||:.....||||||||||:|.|::|::.||||:||::||:.|:
1I6K   200 PKAYITLLDDAKTIEKKIKSAVTDSEGTIRYDKEAKPGISNLLNIYSTLS    249

P67592 251 DMPIKDIEAKYEGEGYGKFKGDLAEIVKAFLVEFQEKYESFYNSDKLDDI    300
           ...|:::|.:|||:|||.||.|||::|...|...||:|..:..|::||.:
1I6K   250 GQSIEELERQYEGKGYGVFKADLAQVVIETLRPIQERYHHWMESEELDRV    299

P67592 301 LDQGRDKAHKVSFKTVKKMEKAMGLGRKR    329
           ||:|.:||::|:.:.|:|||:||||||:|
1I6K   300 LDEGAEKANRVASEMVRKMEQAMGLGRRR    328
```

Figure S2. Sequence alignment between the primary sequence of P67592 and PDB 1I6K. Conserved residues are depicted with a vertical line between the two sequences. Following the BLASTP (Basic Local Alignment Search Tool) analysis using the two sequences, the amino acids involved in catalyzing the reaction are depicted in red.

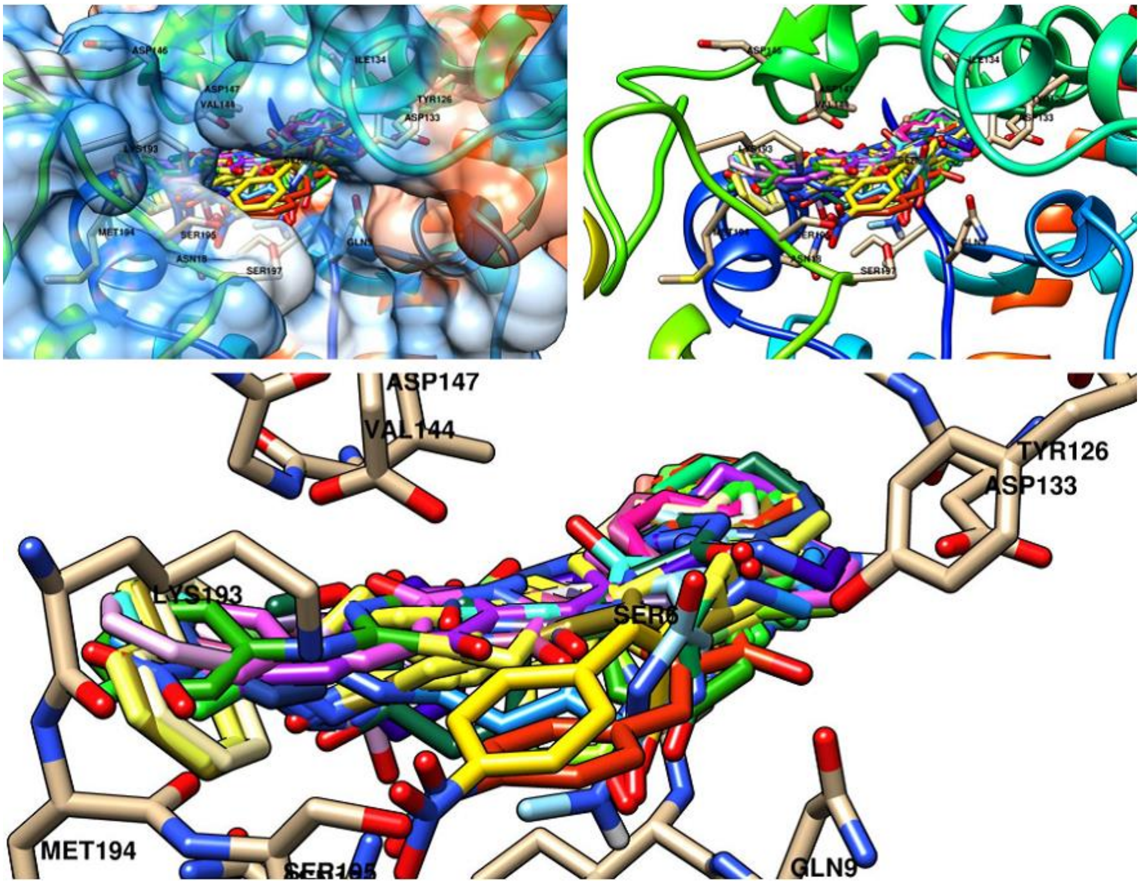

Figure S3. Docking poses of the screened compounds in the active site of *S. aureus* TrpRS: top left image – the target is depicted as thin sticks with secondary structure drawn as ribbon, with simulation of the molecular surface for a better understanding of tridimensional positioning in the active site of enzyme, meanwhile ligands are figured as sticks; top right image - ribbons and bars representation; down image - bars representation

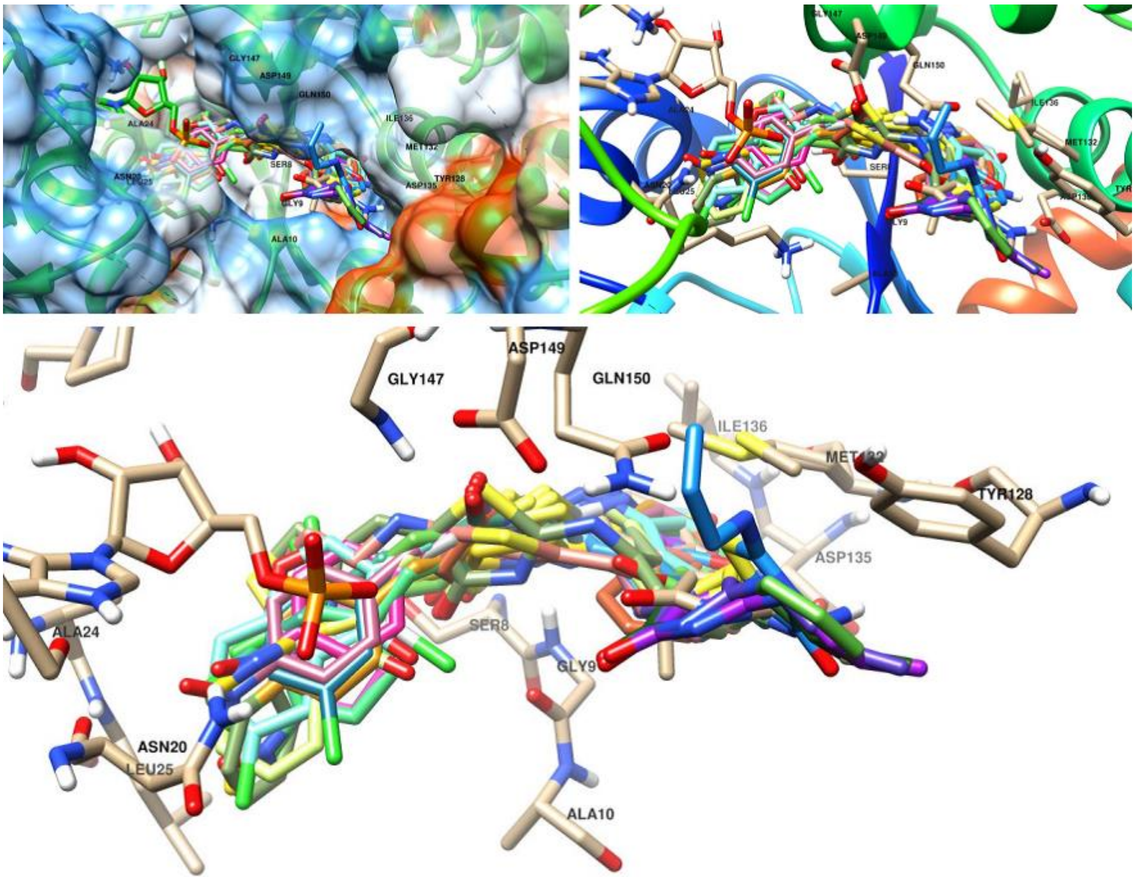

Figure S4. Docking poses of the screened compounds in the active site of *E. coli* TrpRS: top left image – the target is depicted as thin sticks with secondary structure drawn as ribbon, with simulation of the molecular surface for a better understanding of tridimensional positioning in the active site of enzyme, meanwhile ligands are figured as sticks; top right image - ribbons and bars representation; down image - bars representation

The sequence alignment between the primary sequence of P67592_1I6K (*S. aureus*) and that of PDB 5V01 (*E. coli*) revealed a certain degree of similitude between these two structures, but also some significant differences (Figures S5, S6 and Table T1). As results of the alignment between the two sequences, EMBOSS Stretcher returned the following scores: identity=49.2% (164/333), similarity=66.1% (220/333) and gaps=5.1% (17/333). A degree of conservation between the two structures can be found, but some mutations can be ascertained, even in the catalytic site – the 8th amino acid is isoleucine in P67592_1I6K (*S. aureus*) and alanine in 5V01 (*E. coli*). We can

conclude that, strictly regarding the primary structure, there are significant differences between the two enzymes.

```
P67592_1I6K   1 METLFSGIQPSGIPTIGNYIGALKQFVDVQNDYDCYFCIVDQHAITMPQD     50
                ...:|||.|||  .|||||:|||:|:|::|:||.|.:|||||||||:.||
5V0I          1 KPIVFSGAQPS--LTIGNYMGALRQWVNMQDDYHCIYCIVDQHAITVRQD     48

P67592_1I6K  51 RLKLRKQTRQLAAIYLASGIDPDKATLFIQSEVPAHVQAGWMLTTIASVG    100
                ..||||.|....|:|||.||||:|:|:|:||.||.|.|.||.|......|
5V0I         49 AQKLRKATLDTLALYLACGIDPEKSTIFVQSHVPEHAQLGWALNCYTYFG     98

P67592_1I6K 101 ELERMTQYKDKAQKAVEGIPAGLLTYPPLMAADIVLYNTNIVPVGDDQKQ    150
                ||.|||         .|.|.|||..||.||||||:||.||:||||:||||
5V0I         99 ELSRMT---------AENINAGLFDYPVLMAADILLYQTNLVPVGEDQKQ    139

P67592_1I6K 151 HIELTRNLVDRFNSRYNDVLVKPEIRMPKVGGRVMSLQDPTRKMSKSDDN    200
                |:||:|::..|||:.|.|:...||..:||.|.|||||.:||:||||||||
5V0I        140 HLELSRDIAQRFNALYGDIFKVPEPFIPKSGARVMSLLEPTKKMSKSDDN    189

P67592_1I6K 201 AKNFISLLDEPNVAAKKIKSAVTDSDG--IIKFDRDNKPGITNLISIYAG    248
                ..|.|.||::|....||||.||||||.  ::::|..||.|::||:.|.:.
5V0I        190 RNNVIGLLEDPKSVVKKIKRAVTDSDEPPVVRYDVQNKAGVSNLLDILSA    239

P67592_1I6K 249 LTDMPIKDIEAKYEGEGYGKFKGDLAEIVKAFLVEFQEKYESFYNSDK-L    297
                :|...|.::|.::||:.||..||::|:.|...|.|.||:|..|.|.:. |
5V0I        240 VTGQSIPELEKQFEGKMYGHLKGEVADAVSGMLTELQERYHRFRNDEAFL    289

P67592_1I6K 298 DDILDQGRDKAHKVSFKTVKKMEKAMGL---GR    327
                ..::..|.:||...:.:|:|.:.:|:|.   ..
5V0I        290 QQVMKDGAEKASAHASRTLKAVYEAIGFVAKPX    322
```

Figure S5. Sequence alignment between the primary sequences of P67592_1I6K (*S. aureus*) and PDB 5V01 (*E. coli*). Conserved residues are depicted with a vertical line between the two sequences. Following the BLASTP analysis using the two sequences, the amino acids involved in catalyzing the reaction are depicted in red.

The enzyme 1I6K_P67592 of *S. aureus* has a smaller catalytic site compared to PDB 5V0I from *E. coli*, both in terms of volume (966.98 Å^3 vs. 1541.63 Å^3), inner surface (1043 Å^2 vs. 1810.80 Å^2) and depth (27.73 Å vs. 30.29 Å). It has a “Y”-shape, with the distal region heading to the outside of the enzyme, while the *E. coli* enzyme has multiple pockets, with ramifications from its center of catalytic site. The existence of multiple branches can lead to the existence of other sites with unknown functions,

besides the catalytic site. The effect of binding ligands in these pockets may be difficult to predict regarding the possibility and the manner in which these bindings might affect the enzyme activity modulation.

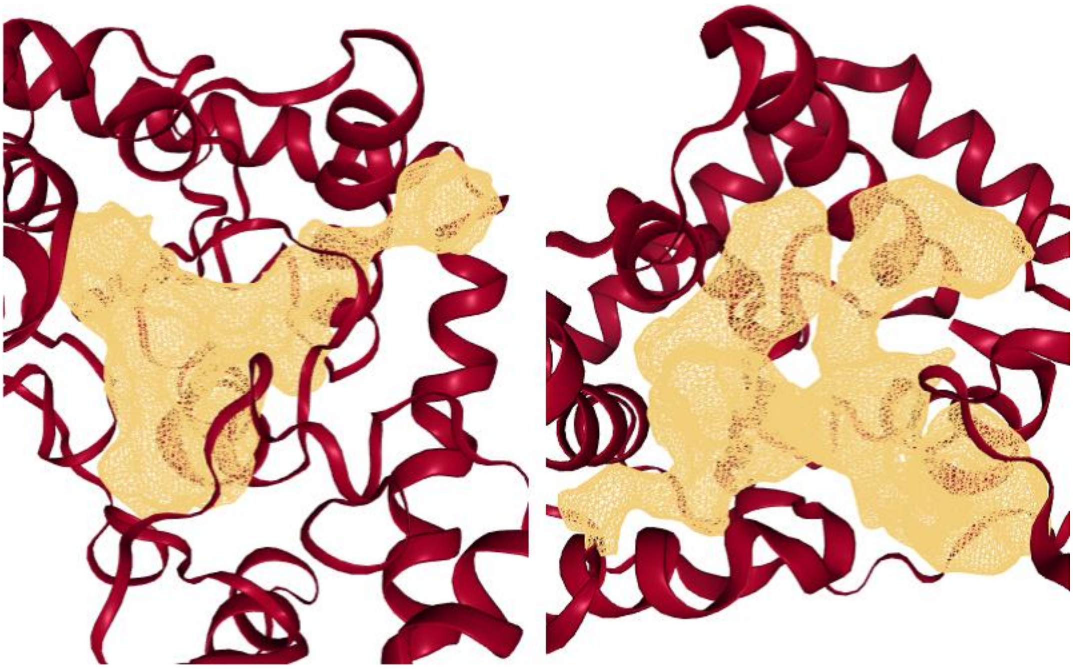

Figure S6. The internal accessible volume in the catalytic site of tryptophanyl-tRNA synthetases used in the molecular docking study. Binding pockets of 1I6K_P67592 from *S. aureus* (left) and of PDB 5V0I from *E. coli* (right) are depicted in yellow mesh.

Table T1. Comparative description of the two tryptophanyl-tRNA synthetases

**Parameter**	**Enzyme binding pocket**	
	**1I6K_P67592 (*S. aureus*)**	**PDB 5V0I (*E. coli*)**
**Volume (Å^3)**	966.98	1541.63
**Surface (Å^2)**	1043.82	1810.80
**Depth (Å)**	27.73	30.29
**Hydrogen bond donors**	31	39
**Hydrogen bond acceptors**	65	85
**Hydrophobic interactions**	28	76
**Hydrophobicity ratio**	0.23	0.38
**Non-polar amino acid ratio**	0.37	0.50
**Polar amino acid ratio**	0.43	0.37
**Positive amino acid ratio**	0.14	0.10
**Negative amino acid ratio**	0.06	0.03
